# Primary esophageal Burkitt’s lymphoma: a rare case report and review of literature 

**Published:** 2014

**Authors:** Irappa Madabhavi, Apurva Patel, Swaroop Revannasiddaiah, Mukesh Choudhary, Asha Anand, Priyanka Das, Harsha Panchal, Sonia Parikh, Suhas Aagre, Vishalkumar Bhardava, Avinash Talele

**Affiliations:** 1 *Department of Medical and Pediatric Oncology, GCRI, Ahmedabad, Gujarat, India *; 2 *Department of Radiation Oncology, Swami Rama Cancer, Hospital & Research Institute, Government Medical College- Haldwani, Nainithal, Uttarakhand, India*; 3 *Department of Radiology, GCRI, Ahmedabad, Gujarat, India *

**Keywords:** Gastrointestinal (GI) lymphoma, Non-Hodgkin’s Lymphoma (NHL), Burkitt’s lymphoma (BL), Diffuse large B-cell lymphoma (DLBCL), COPADM

## Abstract

Esophageal lymphoma is a rare condition, accounting for less than 1% of all gastrointestinal lymphomas. Primary extra nodal esophageal lymphoma constitutes less than 0.2% cases of the total esophageal lymphomas. The definition of primary GI lymphoma has differed among authors. The etiology of the disease is unknown, with the role of Epstein-Barr virus being controversial. The common symptoms of patients with esophageal lymphoma include dysphasia, odynophagia, weight loss, chest pain or present as a result of complications. Burkitt’s lymphoma is one of the fastest growing human malignancies, with a 100% replication rate. Endemic, sporadic (non-endemic) and immunodeficient variants have been recognized. The diagnosis of Burkitt’s lymphoma relies on morphologic findings, immunophenotyping results, and cytogenetic features. Burkitt’s lymphoma is usually treated with LMB-96 protocol depending on the risk stratification. We present a case of primary esophageal Burkitt’s lymphoma, which has been successfully treated with LMB-96 protocol. An extensive review of literature did not reveal a single case of esophageal Burkitt’s lymphoma. To the best of our knowledge this is the first case report in the world literature with diagnosis of primary esophageal Burkitt’s lymphoma.

## Introduction

 Gastrointestinal (GI) tract is the most common extra nodal site involved by lymphoma accounting for 5-20% of all cases (1). Gastrointestinal lymphoma is usually secondary to the widespread nodal diseases. However, primary gastrointestinal lymphoma is very rare, constituting only about 1-4% of all gastrointestinal malignancies. Esophageal lymphoma is a rare condition, accounting for less than 1% of all gastrointestinal lymphomas. 

Primary esophageal lymphoma constitutes less than 0.2% of cases of the total esophageal lymphomas. Primary esophageal lymphoma arises *de novo* in esophagus. Secondary esophageal lymphomas develop by either direct extension or metastasis. The majority of lymphomas involving the esophagus are thought to represent secondary involvement by an adjacent site. Most of the esophageal lymphomas originate from mature B cells and mostly are of diffuse large B-cell lymphoma (DLBCL) and extra nodal marginal zone B-cell lymphoma variety. Primary esophageal Burkitt's lymphoma (BL) is one of the rarest varieties of the non-Hodgkin’s lymphoma (NHL) of GI tract encountered in day to day clinical practice. Extensive search of the literature couldn’t reveal a single case of primary esophageal BL. 

## Case Report

**Figure 1:**
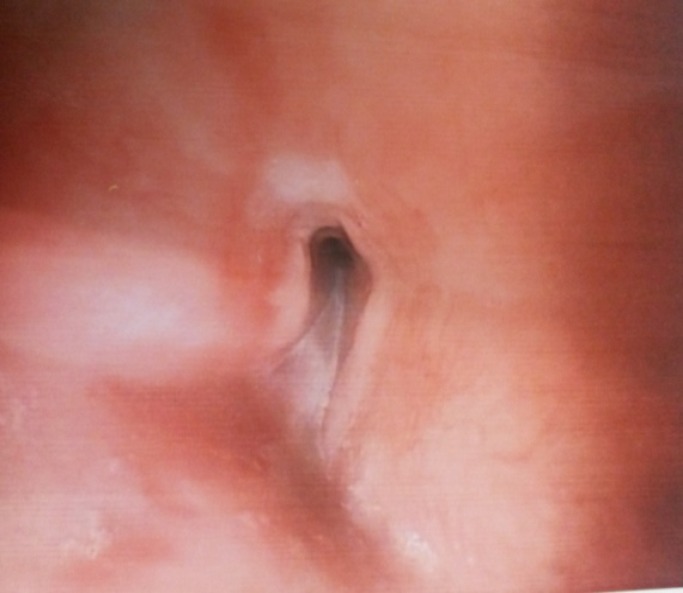
Shows complete obstruction of the esophagus at the level of the mid esophagus around 25cm from incisors, lining mucosa appears normal without any ulceration or growth and scope couldn’t be negotiated beyond the obstruction

Seventeen-year-old lady presented to a local physician with the symptoms of progressive dysphasia, initially for solids followed by liquids, over a period of 2 weeks. There was no history suggestive of any acid or lye ingestion in the past. There was no history suggestive of any lympadenopathy, weight loss, fever, and night sweats. On endoscopic examination there was a complete obstruction of the esophagus at the level of the mid esophagus around 25cm from incisors, lining mucosa appears normal without any ulceration or growth and scope couldn’t be negotiated beyond the obstruction (Figure 1). 

**Figure 2:**
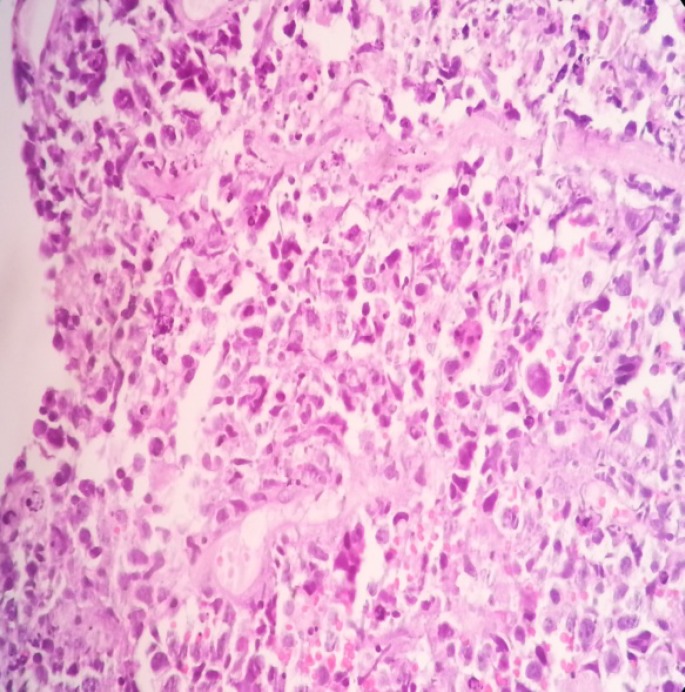
CT image showing circumferential homogeneously enhancing smooth walled upper esophageal thickening of around 15mm

Computed tomography (CT) scan of the thorax shows circumferential homogeneously enhancing smooth wall thickening, involving upper esophagus, without any associated mediastinal lympadenopathy or mass lesion (Figure 2). 

Endoscopic ultrasonographic features suggest the presence of hypoechoic thickening of the muscularis mucosa and submucosa without any evidence of periesophageal nodes.

 Endoscopic fine needle aspiration cytology smears reveal mainly mature lymphocytes with a few atypical cells in the background of granular necrotic debris. In view of high prevalence of the tuberculosis in under developing countries patient was put on anti-tubercular drugs with endoscopic esophageal dilatation by the local physician. After one week, patient again presented with similar complaints without any symptomatic relief. Patient was re-subjected to endoscopic dilatation and anti-tubercular drugs were continued. 

**Figure 3:**
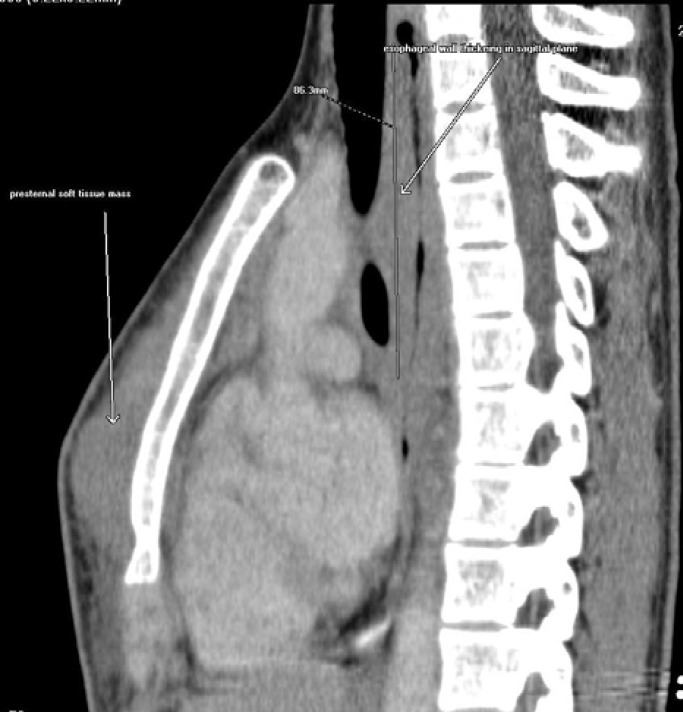
Sagittal CT image shows gross circumferential thickening with luminal narrowing involving upper and mid esophagus with a soft tissue mass lesion of size 10×6×2cm in anterior chest wall in left para sternal region Figure 3. Sagittal CT image shows gross circumferential thickening with luminal narrowing involving upper and mid esophagus with a soft tissue mass lesion of size 10×6×2cm in anterior chest wall in left para sternal region

Patient was referred to our cancer care center by the local physician for expert opinion. On examination her height, weight, and body mass index were within normal range for her age. There was no pallor, lympadenopathy, spleenomegaly, hepatomegaly, or any local mass in the abdomen. Her routine investigations revealed hemoglobin of 11.5gm/dl, total count of 9600/cu mm and platelet count of 326000/cu.mm. Erythrocyte sedimentation rate (ESR) was 70mm, Mantoux test was normal, uric acid was 6.8mg/dl, creatinine was 0.7mg/dl, potassium was 4.2mg/dl, and lactate dehydrogenase was 150IU/L. Serology for human immunodeficiency virus, Epstein-Barr virus (VCA-IgG, IgM, and EBV nuclear antigen-IgG) and hepatitis B and C viruses were negative. Bone marrow aspiration and trephine biopsy cytology didn’t reveal any lymphomatous infiltration. Cerebrospinal fluid analysis was normal. On further imaging with repeated CT scan of thorax shows gross circumferential thickening with luminal narrowing involving upper and mid esophagus with a soft tissue mass lesion of size 10×6×2cm in anterior chest wall in left para sterna region without any other abnormality (Figure 3). CT scan of abdomen and pelvis didn’t reveal any mass or lympadenopathy. 

**Figure 4:**
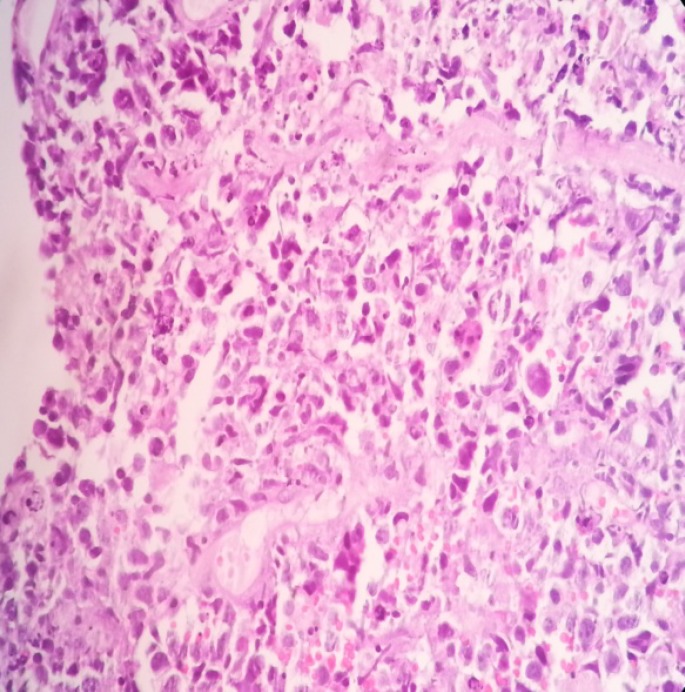
Shows malignant cells arranged in sheets, round in shape, pleomorphic nuclei, medium to large in size with prominent nucleioli, with fine chromatin and moderate cytoplasm. Also shows many mitotic figures and apoptotic bodies (starry sky pattern); H & E staning; x40

Histopathological examination of the CT scan guided biopsy specimen shows mature B cells arranged in sheets, round in shape, pleomorphic nuclei, medium to large in size with prominent nucleioli, with fine chromatin and moderate cytoplasm with many mitotic figures (Figure 4). 

**Figure 5:**
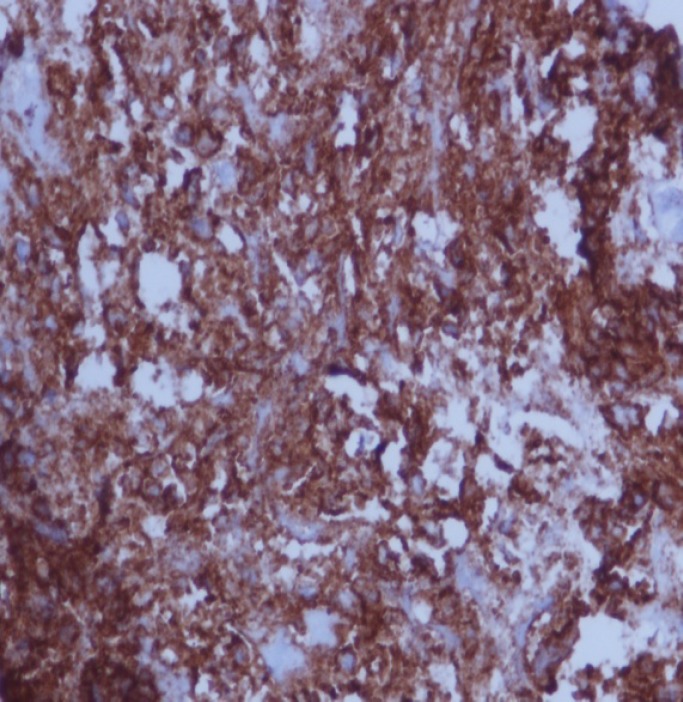
The immunophenotypic picture of the specimen shows positivity to B-cell antigen CD20; IHC; x40

**Figure 6:**
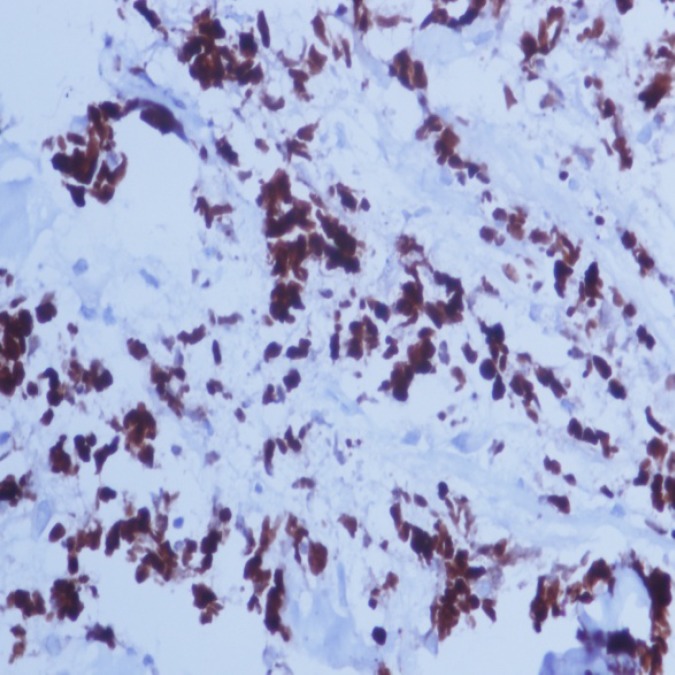
Shows the high proliferation rate with numerous mitotic figures and apoptotic bodies. Nearly all cells (>99%) expressed proliferation antigen Ki-67, which is recognized by the antibody MIB-1; IHC; x40

Numerous apoptotic bodies were also seen, which are engulfed by the macrophages imparting the characteristic “starry sky” appearance. The immunophenotypic picture of the specimen shows negativity to BCL-2 and positivity to B-cell antigens CD19, CD20 (Figure 5), CD22 and CD10 and BCL-6. The proliferation rate is extremely high with numerous mitotic figures and apoptotic bodies. Nearly all cells (>99%) expressed proliferation antigen Ki-67, which is recognized by the antibody MIB-1 (Figure 6).

Staging evaluation revealed a stage II intermediate risk, Group-B disease by Murphy staging system. Patient was managed with supportive care for tumor lysis syndrome prevention and LMB-96 protocol for intermediate risk BL with COP [cyclophosphamide, oncovin (vincristine) and prednisolone] as reduction or prophase, COPADM [cyclophosphamide, oncovin (vincristine), prednisolone, doxorubicin and high dose methotrexate (HDMTX)] 1 & 2 as induction chemotherapy and CYM (cytarabine, HDMTX) 1 & 2 as consolidation chemotherapy courses with intrathecal (IT) chemotherapy prophylaxis during all phases of the therapy.

COP →COPADM1→ COPADM2→ CYM1→CYM2.

**Figure 7:**
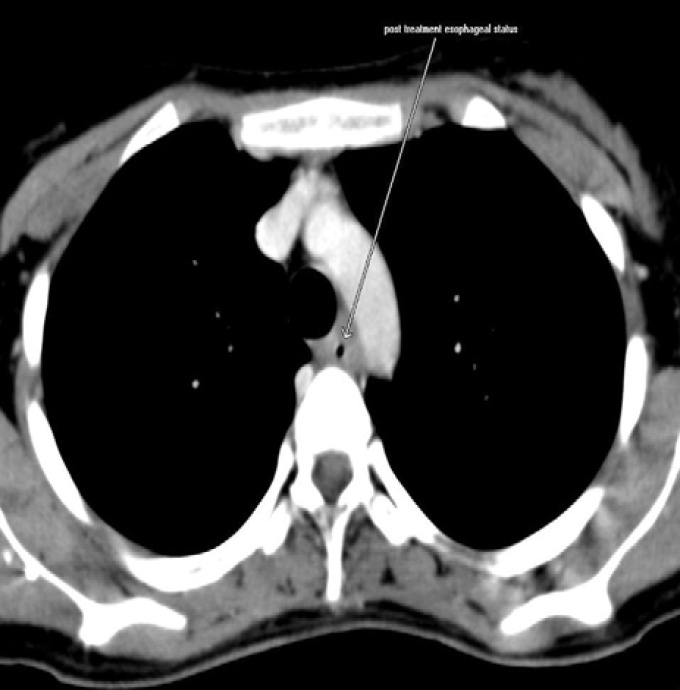
Post-treatment CT image shows complete disappearance of the left parasternal mass with normal esophageal thickening

The first evaluation was performed after COP at day 7 with a response rate of more than 20%. The second evaluation was performed after first COPADM with a response rate of more than 50% decrease in the size of mass and normal upper GI endoscopy. The third evaluation was performed after first CYM with CT image showing complete response rate without any residual lesion with normal esophageal thickening (Figure 7). Patient is under regular follow up in our tertiary care center with imaging reports for any recurrence since 9 months. 

## Discussion

Most non-Hodgkin’s lymphomas arise in lymph nodes or other lymphatic tissues such as the spleen, Waldeyer’s ring and thymus. Involvement of so-called extra nodal organs is a common finding after staging investigation. A substantial part of NHL even arises in extra nodal sites, often referred to as primary extra nodal NHL. Twenty five to forty percent of NHL patients present with a primary extra nodal lymphoma (2).

The gastrointestinal tract is the predominant site of extra nodal NHL involvement (3). Primary lymphomas of the GI tract are rare, while secondary GI involvement is relatively common. The definition of primary GI lymphoma has differed among authors. 

There are at least two deﬁnitions of primary GI NHL in use. The one by Dawson et al. (4) is restricted to localized disease (stages IE, IIE), whereas the study by Lewin et al. (5) requires that patients exhibit GI symptoms or predominant lesions in the GI tract. Dawson et al. (4) developed the following 5 criteria needed to identify a localized primary GI lymphoma.

- No palpable superficial lymph nodes
- Normal chest radiograph findings with no evidence of lympadenopathy
- Normal white blood cell (WBC) count
- Predominant lesion within the GI tract, with lymph node involvement confined to the lymph node chain involved in drainage of that specific GI segment
- No involvement of the liver or spleen

Overall, the stomach is the most common site of involvement in primary gastrointestinal lymphomas, followed by the small intestine. The vast majority of gastrointestinal lymphomas are B-cell lymphomas. T-cell lymphomas occur mostly in the small intestine, associated with longstanding celiac disease. Most common ones are diffuse large B-cell lymphoma and marginal zone lymphoma of mucosa-associated lymphoid tissue (MALT). Other B-cell lymphomas are Burkitt’s lymphoma, mantle cell lymphoma, and follicular lymphoma. Only 1-2% of adult non-Hodgkin’s lymphomas are Burkitt’s lymphoma.

Although lymphoma may involve any part of the gastrointestinal tract either primarily or secondarily, esophageal involvement is rare and accounting for only less than 1% of all gastrointestinal lymphomas. The majority of lymphomas involving the esophagus are thought to represent secondary involvement by an adjacent site and usually results from metastasis from the cervical or mediastinal lymph nodes or extension of a gastric lymphoma (6). 

Primary esophageal lymphoma is an extremely rare occurrence, with fewer than 30 cases reported in the literature, with the majority being diffuse large B-cell type non-Hodgkin lymphomas. Non-lymphoid malignancy or systemic lymphoma should be ruled out before making a diagnosis of primary esophageal lymphoma. The age of presentation of the disease is highly variable. The etiology of the disease is unknown, with the role of Epstein-Barr virus being controversial. To the best of our knowledge Burkitt’s lymphoma of the esophagus is not yet reported in medical literature. 

Burkitt’s lymphoma is one of the fastest growing human malignancies, with a 100% replication rate and a doubling time of approximately 25 hours. Burkitt’s lymphoma is a high grade B-cell neoplasm under the umbrella of non-Hodgkin’s lymphomas and is seen predominantly in children’s and young adults, and to a lesser extent in middle-aged adults. Endemic, sporadic (non-endemic) and immunodeficient variants have been recognized (7).

The endemic (African) variant most commonly occurs in children living in malaria endemic regions of the world. Epstein-Barr virus (EBV) infection is found in nearly all patients. It is believed that chronic malaria reduces resistance to EBV, allowing it to take hold. The disease characteristically involves the jaw or other facial bones. Sporadic lymphomas are rarely associated with the Epstein-Barr virus. Majority of sporadic BL present with abdominal masses, whereas jaw is rarely affected; majority of BL patients (70 %) present at advanced stages (8). Immunodeficiency-associated Burkitt’s lymphoma is usually associated with HIV infection or post-transplant patients who are taking immunosuppressive drugs.

The common symptoms of patients with esophageal lymphoma include dysphasia, odynophagia, weight loss, chest pain, or present as a result of complications such as hemorrhage, obstruction, or perforation with a trachea-esophageal fistula. Constitutional B symptoms (fever and night sweats) are not typically present. The diagnosis of Burkitt’s lymphoma relies on morphologic findings, immunophenotyping results, and cytogenetic features (9).

Notably the histological hallmark of BL is the presence of numerous apoptotic cells within scattered pale phagocyte macrophages, a feature responsible for the “starry sky” microscopic appearance of the cells and is formed by the phagocyte histiocytes interspersed among primitive round monomorphic and deeply basophilic lymphoblasts. The tumor cells in BL generally strongly express markers of B cell differentiation (CD20, CD22, and CD19) as well as CD10, and BCL6. The tumor cells are generally negative for BCL2 and TdT. The high mitotic activity of BL is confirmed by nearly 100% of the cells staining positive for Ki67 (10).

The translocation of the *c-myc* gene and its consequent deregulation is a key oncogenic event in the development of Burkitt’s lymphoma that contributes to lymphoma genesis through alterations in cell cycle regulation, cellular differentiation, apoptosis, cellular adhesion, and metabolism. Accordingly, the signature of the *c-myc* target genes distinguished BL from diffuse large-B-cell lymphoma. However, *c-myc* translocations also occur in 5 to 10 percent of diffuse large-B-cell lymphomas. Burkitt’s lymphoma and diffuse large-B-cell lymphoma were found to differ with respect to the signature of the *c-myc* target genes as well as the other three gene-expression signatures like “BL-high” signature, major-histocompatibility-complex (MHC) class I genes, and nuclear factor-κB (NF-κB) target genes (11). The most common translocation variant is t(8;14) (q24;q32), which accounts for approximately 85% of cases. The other two less-common translocations, t(2; 8) (p12; q24) and t(8; 22) (q24; q11), account for the remaining 15% of cases (12).

Because of the frequency of extra nodal disease, several different staging systems have been used for BL like Ann Arbor system and St. Jude or Murphy staging schema. Risk classification was defined as low risk (group A) with resected stage I and abdominal completely resected stage II, high risk (group C) with bone marrow (BM) involvement (L3 blasts ≥25% and/or CNS disease), and intermediate risk (group B) was all others.

Our patient fits into the intermediate risk (group B). Group B patients should be managed with 7-day, low-dose, prophase cyclophosphamide, vincristine, and prednisone (COP) therapy. Induction therapy consisted of two cycles of fractionated COPADM (cyclophosphamide, oncovin, vincristine, prednisolone, doxorubicin, and high-dose methotrexate 3g/m^2^). Patients then continued with two consolidation cycles of cytarabine and HD-MTX (CYM) and should be concluded with one maintenance phase of COPADM (COPADM-3) with IT chemotherapy prophylaxis during all phases of the therapy. Patients with less than a 20% response on day 7 of COP and patients with residual disease after CYM-1 should be transferred to rescue group C therapy (13).

Burkitt’s and B-cell lymphomas in childhood have an excellent overall prognosis regardless of the location [except for primary central nervous system (CNS) lymphoma], especially when treated with contemporary chemotherapy protocols (14). In non-resected mature B-cell Burkitt’s lymphoma of childhood and adolescence intermediate risk group with no CNS involvement, a response after prophase COP and a CR obtained after the first consolidation course, a 90% cure rate can be achieved with an intensive but shortened treatment delivering low doses of cyclophosphamide (3.3gm/m^2^) and doxorubicin (120mg/m^2^) (D12). All the treated patients should be kept under regular follow up disease site imaging initially once a month for 6 months then once in three months. 

In conclusion, primary esophageal BL is one of rarest variety of gastrointestinal extranodal lymphoma. Burkitt’s lymphoma is one of the fastest growing human malignancies, with a 100% replication rate. Accurate early diagnosis with imaging, histopathological and immunohistochemical features are utmost important in the proper management of the patient. An extensive review of literature did not reveal a single case of esophageal BL. To the best of our knowledge this is the first case report in the world literature with diagnosis of primary esophageal Burkitt’s lymphoma.

**Learning Points**

1. Primary lymphoma of the esophagus should be kept in mind as one of rarest cause of the esophageal stricture.
2. Charecterstic imaging, histopathological, and immunohistochemical features helps in the diagnosis of the esophageal BL.
3. Most commonly used staging systems are Ann Arbor system and St. Jude or Murphy staging schema.
4. Best managed with LMB-96 protocol.
5. Burkitt’s and B-cell lymphomas in childhood have an excellent overall prognosis.
